# Breastfeeding monitoring in Germany – What contribution can the data from KiGGS provide?

**DOI:** 10.17886/RKI-GBE-2016-044

**Published:** 2016-12-14

**Authors:** Anna-Kristin Brettschneider, Cornelia Weikert, Klaus Abraham, Franziska Prütz, Elena von der Lippe, Cornelia Lange

**Affiliations:** 1 Robert Koch Institute, Department for Epidemiology and Health Monitoring, Berlin, Germany; 2 Federal Institute for Risk Assessment, Food bafety Department, Berlin, Germany

**Keywords:** BREASTFEEDING BEHAVIOUR, BREASTFEEDING PROMOTION, BREASTFEEDING MONITORING IN GERMANY, HEALTH SURVEY, TEMPORAL TRENDS

## Abstract

A continuous breastfeeding monitoring is essential as it enables reports on changes in breastfeeding behaviour. The German Health Interview and Examination Survey for Children and Adolescents (KiGGS), which is conducted by the Robert Koch Institute, periodically collects data about the health of children and young people living in Germany, including data on breastfeeding. Moreover, KiGGS is mentioned within the approach developed by the National Breastfeeding Committee as a possible source of data for breastfeeding monitoring.

The data from KiGGS can be used to develop retrospective indicators on breastfeeding for particular birth cohorts. The data demonstrate that the prevalence of children who were ever breastfed tended to rise between the 2001/2002 and 2007/2008 cohorts; however, no significant changes were identified for the 2001–2008 cohorts with respect to breastfeeding duration. Breastfeeding monitoring relies on reports about current trends in the field; due to the periodicity with which the KiGGS study waves are conducted, data on current birth cohorts cannot be provided. Therefore, data on breastfeeding needs to be collected throughout Germany in relation to direct environmental and other factors. This data should be collected during health screenings and regular check-ups so that it can be used as a further measure in breastfeeding monitoring

## 1. Introduction

Breastfeeding is linked to many short-term and long-term benefits for the health and capacities of breastfed children; it helps ensure that they grow up healthily and contributes to the prevention of various diseases. In the short-term, fewer gastrointestinal and respiratory infections are observed among breastfed children [1]. Furthermore, the results of a recent meta-analysis suggest that breastfeeding is associated with a long-term reduction in the risk of becoming overweight and of obesity, as well as with a slightly higher intelligence quotient [2]. Breastfeeding also strengthens the bond between mother and child.

The World Health Organisation (WHO), along with other international organisations, actively supports the promotion of breastfeeding. According to the Global Strategy for Infant and Young Child Feeding, during the first six months infants should be exclusively breastfed to achieve optimal growth, development and health [3]. At the European level, the Global Strategy was used to establish an action plan entitled Protection, Promotion and Support of Breastfeeding in Europe [4]. This action plan provides guidelines for the development and implementation of measures aimed at promoting breastfeeding in Europe. A call for standardisation in breastfeeding monitoring constitutes an essential component of the action plan. The plan defines breastfeeding monitoring as the systematic collection of current, comprehensive and accurate data on breastfeeding rates and breastfeeding behaviour at the national and regional level. The German National Breastfeeding Committee has prepared a plan to monitor breastfeeding in Germany [5]. The aim is to adopt an integrative approach that will enable various data sources to be analysed together, while providing complementary results. This should help provide a complete and up-to-date picture of breastfeeding and the conditions in which breastfeeding takes place. Moreover, it could also help establish targeted planning measures that promote breastfeeding and study their effectiveness.

**Fig. 1:**
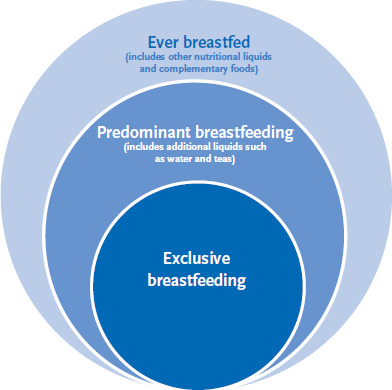
Definitions of breastfeeding Source: Own diagram based on [12, 13]

The German Health Interview and Examination Survey for Children and Adolescents (KiGGS), which is conducted by the Robert Koch Institute, is listed as a data source for integrated breastfeeding monitoring within the approach developed by the National Breastfeeding Committee. Results on breastfeeding from the KiGGS study have already been published and enable overall assessments of breastfeeding rates in Germany over longer periods (birth cohorts 1996–2002 and 2002–2012) [6, 7]. Consequently, a detailed analysis of the data in the KiGGS Baseline Survey and KiGGS Wave 1 is important as it would provide extremely useful data for breastfeeding monitoring. Furthermore, it would also enable reports to be made about developments in breastfeeding rates by cohort, and about breastfeeding duration (whether a child was breastfed for two, four, six months, or longer). In addition, it would also enable the study of whether a child was ever, exclusively or predominantly breastfed (Figure 1). This analysis also aims to understand the contribution that the KiGGS data could make to breastfeeding monitoring at the national level in Germany.

## 2. Methods

KiGGS is a combined cross-sectional and cohort study (for more details about the methodology used see [8–10]). As part of the KiGGS Baseline Survey (2003–2006), a total of 17,641 children and adolescents were studied at 167 sample points (response rate: 66.6%). The follow-up survey, KiGGS Wave 1 (2009–2012), involved a cross-sectional sample of 4,455 newly invited participants aged 0–6 years and 7,913 re-invited participants aged 7–17 years (response rate: 38.8% for newly invited participants; 72.8% for re-invited). In the KiGGS Baseline Survey data on breastfeeding behaviour was gathered from all parents with children and adolescents aged 0 to 17, while in KiGGS Wave 1 from parents of children aged 0 to 10. This means, the data on breastfeeding was gathered retrospectively from different periods (for more details about the collection of breastfeeding data see [7, 11]). If data was gathered in both waves and there was a discrepancy in the answers, the data from the baseline survey was considered to be the correct one (lower recall bias). If discrepancy in the data was observed in cases where the information came from different respondents, the information given from the mother was considered to be the correct one.

Based on the information provided by parents, data from the KiGGS Baseline Survey and KiGGS Wave 1 for the birth cohorts 2001–2008 were used to ascertain the proportion of children who were breastfed until they were two, four or six months old (or older), and whether a child was ever, predominantly, or exclusively breastfed. The data was also used to calculate the average duration of breastfeeding. Children who are exclusively breastfed do not receive any other liquids or complementary foods in addition to breast milk; in contrast, additional liquids such as water or tea may be provided to predominantly breastfed children (this category also includes children who were exclusively breastfed). Children who were ever breastfed will also have been fed other nutritious liquids (in particular infant formula) and supplementary foods (therefore, this category also includes children who were exclusively or predominantly breastfed) [12, 13] (Figure 1).

Two cohorts were combined for the analyses: 2001/2002, 2003/2004, 2005/2006 and 2007/2008. Since the 2006 Parental Allowances and Parental Leave Act applies to all children born after 1 January 2007, [14] it was also interesting to see whether breastfeeding rates or duration differed between the birth cohorts 2007–2008 from those of previous cohorts. As a significant proportion of the 2009–2012 cohort was still breastfed at the time of the survey (the KiGGS Wave 1 survey period), and it was not clear for how long these children would be breastfed, these cohorts were not taken into account as part of the current analysis.

The duration of exclusively breastfeeding was also stratified according to age and educational status of the mother, number of siblings, smoking during pregnancy, (pre)maturity and place of residence. Educational status was categorised in accordance with the international form of classification set out as part of the Comparative Analyses of Social Mobility in Industrial Nations (CASMIN) [15, 16]. Since no differences between girls and boys were found in terms of breastfeeding rates, [7] a gender-based analysis was not conducted.

## 3. Results

The KiGGS data demonstrate an increase in the prevalence of breastfeeding in Germany: whereas 77.0% of children from the 2001/2002 cohorts were ever breastfed, this rate increased to 82.5% among the 2007/2008 cohorts. At the age of six months, about half of the infants were still being breastfed in all cohorts. After this point, however, breastfeeding rates decreased significantly (Table 1).

**Fig. 2:**
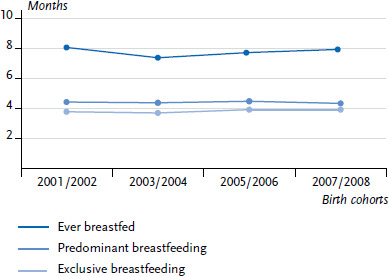
Average duration of ever breastfed, predominant and exclusive breastfeeding, by cohort (based on all the children who were ever breastfed, n = 4,324)) Source: KiGGS Baseline Study (2003–2006); KiGGS Wave 1 (2009–2012))

Two-thirds (66.4%) of the children from the 2007/2008 cohorts were exclusively breastfed, even if it was for a short time. The rate was 63.4% for the 2001/2002 cohorts. The present analyses of KiGGS data show that the prevalence of exclusive breastfeeding between birth and a child’s second month of life only decreased slightly (by about five to eight percentage points). However, the prevalence of exclusive breastfeeding decreased particularly between the second and fourth month, with an average difference of 22 percentage points (Table 1). The average duration of exclusive breastfeeding was about four months (Figure 2).

A significant decline in predominant breastfeeding was also identified for children between two and four months. At two months, 64.6% of children born in 2007/2008 were predominantly breastfed; even at four months, the rate was only 48.9% (Table 1). Rates of exclusive and predominant breastfeeding and the average duration of breastfeeding remained almost constant for the 2001–2008 cohorts (Figure 2).

Regarding determinants of breastfeeding, the children of mothers with lower levels of education were exclusively breastfed for a significantly shorter period than children of mothers with medium to higher levels of education (Table 2). In addition, children of mothers who smoked during pregnancy, as well as infants who were born prematurely, were breastfed for a significantly shorter period. There is also a positive correlation between the mother’s age and the duration of breastfeeding among all cohorts: the duration of exclusive breastfeeding increases with the age of the mother. There was no difference with regards to place of residence (in eastern or western Germany).

## 4. Discussion

A comparison of the KiGGS results with other studies demonstrates that KiGGS shows a slightly lower rate of ever breastfed than the rates reported from regional studies [17]. However, the rate identified by KiGGS is similar to the nationwide online survey conducted by Libuda et al. (2014). This study showed that 78% of the children from birth cohorts 2007–2010 were ever breastfed [18]. The differences between the findings of regional studies can be explained by the different methodological approaches they used (for example, a prospective study design or the exclusion of prematurely born children). In addition, recall bias also plays a role in retrospective studies, and breastfeeding that lasted for a very short period may be assessed differently in retrospect. Therefore, direct comparisons with other studies are limited.

The rates of predominant breastfeeding at the age of two months from the KiGGS 2007/2008 cohorts (64.6%) are relatively close to the results of Jöllenbeck et al. (2012) for 2008/2009 (65%). However, these data are from a study with regional limitations [19]. The rates of predominant breastfeeding at the age of four months among the 2007/2008 cohorts (48.9% and 50%) also are confirmed by both studies.

The relatively slight decline in the rate of breastfeeding between birth and two months among the KiGGS data was not confirmed by other (prospective) studies. According to these studies, despite an initially high rate of breastfeeding, the strongest decrease in (predominant) breastfeeding occurs during the first two months of a child’s life [17]. This might be related to the fact that prospective studies also include breastfeeding that occurs only in the first few days after birth; there may be a decline of about 10 percentage points in the first week of a child’s life [19]. It is also possible that short breastfeeding periods such as these are forgotten or even go unmentioned in retrospective studies. Therefore, in retrospective studies a recall bias cannot be excluded. However, studies about the ability to remember with respect to breastfeeding have shown that questions about whether breastfeeding took place at all and on breastfeeding duration do produce valid answers [20, 21]. Nevertheless, there was a recall bias regarding the precise moment of starting complementary feeding [20].

**Infobox: Breastfeeding recommendations**

**Recommendations by the WHO**

“As a global public health recommendation, infants should be breastfed exclusively for the first six months of life to achieve optimal growth, development and health” [4].

**Recommendations by the National Breastfeeding Committee at the Federal Institute for Risk Assessment**

“Breast milk is the best food for nearly all infants. Exclusive breastfeeding during the first six months provides an adequate diet for the majority of infants.

The point at which an infant will need supplementary foods in addition to breastfeeding depends on the child’s well-being and his or her ability to eat. Supplementary foods should be provided no later than at seven months, and no earlier than at five months. The introduction of solid food does not mean that a child should be weaned off breast milk immediately, but instead that the amount of breast milk provided to the child and the frequency of breastfeeding can be reduced. It is up to the mother and the child to jointly decide when it is time to stop breastfeeding” [28].

The current study of KiGGS data showed that external factors such as legislation on parental leave and parental benefits had no discernible influence on breastfeeding behaviour. Nevertheless, in the interest of structural prevention, it would be important to see whether higher breastfeeding rates will be observed in the following years; even though it would not be possible to derive a causal relationship from the data in this case.

The fact that mothers with lower levels of education exclusively breastfeed for a shorter period than mothers with medium or higher education has also been shown by various other national and international studies [22–24]; the same applies to smoking in pregnancy and to premature birth [25]. The positive correlation between maternal age and the duration of exclusive breastfeeding is also demonstrated by other studies [26, 27].

## 5. Conclusions for breastfeeding monitoring

The KiGGS study has the long-term goal of monitoring and reporting on the health of children and in Germany. As breastfeeding is just one aspect of the KiGGS study, information about factors that encourage or impede breastfeeding cannot be recorded in detail due to the study’s scope. However, the data from KiGGS can be used to retrospectively develop indicators for ever, predominant and exclusive breastfeeding, for cohorts for which conclusive data is available. Due to the periodicity of KiGGS waves (about every five years), it is impossible to regularly report on the breastfeeding behaviour of current cohorts. However, breastfeeding monitoring requires current data. Therefore, data on breastfeeding needs to be gathered throughout Germany in relation to direct environmental factors and factors influencing breastfeeding in the context of health screenings for children and regular check-ups. This data could then be used as a further measure that could help develop an overall and up-to-date picture of breastfeeding and the conditions in which it occurs. In turn, and this is foreseen as part of the approach developed on integrative breastfeeding monitoring by the National Breastfeeding Committee [5], this could form a basis with which to plan and review the effectiveness of targeted interventions aimed at increasing breastfeeding rates in Germany.

## Key statements

- Breastfeeding monitoring involves the systematic collection of current, comprehensive and accurate data on breastfeeding rates and behaviour at the national and regional level with the aim of optimally promoting breastfeeding.
- The KiGGS data demonstrate that the proportion of children who have been ever breastfed tended to increase between the 2001/2002 and 2007/2008 cohorts. However, there was no significant change in breastfeeding duration.
- The number of infants who are exclusively or predominantly breastfed significantly falls when children reach two to four months.
- A significant decline can be observed in breastfeeding rates after a child becomes six months old for all categories of breastfeeding.
- Alongside other potential data sources, the data from KiGGS can provide a contribution to breastfeeding monitoring at the national level.

## Figures and Tables

**Table 1:** The prevalence of breastfeeding: exclusive and predominant breastfeeding, and ever breastfed in a child’s life, according to cohort Source: KiGGS Baseline Study (2003–2006); KiGGS Wave 1 (2009–2012)

		Cohort 2001/2002	Cohort 2003/2004	Cohort 2005/2006	Cohort 2007/2008
	n	% (95% CI)	% (95% CI)	% (95% CI)	% (95% CI)
**Exclusive breastfeeding**					
Ever	3,281	63.4 (58.6 – 68.0)	63.1 (58.7 – 67.3)	68.0 (63.2 – 72.4)	66.4 (62.0 – 70.5)
2 months	2,911	55.5 (50.6 – 60.2)	56.4 (51.9 – 60.8)	61.5 (56.7 – 66.1)	58.9 (54.8 – 62.9)
4 months	1,817	33.2 (29.4 – 37.2)	31.5 (28.0 – 35.2)	39.1 (34.9 – 43.4)	38.4 (34.5 – 42.5)
6 months	590	10.2 (8.1 – 12.9)	9.2 (7.2 – 11.7)	12.4 (10.2 – 15.1)	11.9 (9.8 – 14.5)
> 6 months	202	4.1 (2.6 – 6.5)	3.8 (2.6 – 5.6)	3.7 (2.5 – 5.5)	4.4 (3.1 – 6.2)
**Predominant breastfeeding**					
Ever	3,747	70.4 (65.8 – 74.6)	70.7 (66.1 – 74.9)	71.8 (67.1 – 76.0)	72.0 (67.6 – 76.1)
2 months	3,493	65.9 (61.3 – 70.5)	66.7 (62.1 – 70.9)	68.1 (63.3 – 72.6)	64.6 (60.4 – 68.5)
4 months	2,552	44.5 (40.5 – 48.5)	47.3 (42.8 – 51.9)	49.2 (44.8 – 53.6)	48.9 (44.8 – 53.0)
6 months	1,026	20.5 (17.2 – 24.2)	17.3 (14.5 – 20.5)	20.3 (17.3 – 23.6)	18.5 (15.7 – 21.6)
> 6 months	426	9.2 (7.0 – 11.9)	8.0 (6.2 – 10.4)	8.7 (6.6 – 11.2)	8.0 (6.1 – 10.4)
**Ever breastfed**					
Ever	4,324	77.0 (72.6 – 81.0)	80.3 (76.1 – 84.0)	81.5 (77.3 – 85.1)	82.5 (78.6 – 85.8)
2 months	4,092	74.0 (69.5 – 78.1)	73.5 (68.9 – 77.6)	75.1 (70.5 – 79.2)	77.3 (73.2 – 80.9)
4 months	3,429	58.6 (54.2 – 62.9)	59.6 (55.2 – 63.9)	62.2 (57.5 – 66.7)	65.6 (61.3 – 69.6)
6 months	2,909	49.2 (45.0 – 53.3)	49.3 (44.9 – 53.7)	53.3 (48.7 – 58.0)	54.4 (50.0 – 58.7)
12 months	979	17.2 (14.4 – 20.4)	14.9 (12.3 – 17.9)	17.9 (15.0 – 21.3)	21.7 (18.4 – 25.4)
> 12 months	589	11.8 (9.4 – 14.8)	7.8 (6.3 – 9.7)	11.0 (8.7 – 13.8)	12.4 (10.2 – 14.9)

CI = confidence interval

**Table 2:** The duration of exclusive breastfeeding according to subgroups Source: KiGGS Baseline Study (2003–2006); KiGGS Wave 1 (2009–2012)

		Cohort 2001/2002	Cohort 2003/2004	Cohort 2005/2006	Cohort 2007/2008
	n	M in months (95% CI)	M in months (95% CI)	M in months (95% CI)	M in months (95% CI)
**Age of mother**					
≤ 24 years	531	3.47 (2.61 – 4.32)	2.81 (2.38 – 3.25)	3.61 (2.94 – 4.27)	3.41 (2.80 – 4.02)
25 – 29 years	1,409	3.53 (3.13 – 3.94)	3.48 (3.13 – 3.83)	3.74 (3.36 – 4.12)	3.54 (3.18 – 3.89)
30 – 34 years	1,855	3.85 (3.59 – 4.10)	4.20 (3.88 – 4.51)	3.97 (3.63 – 4.31)	4.11 (3.89 – 4.34)
≥35 years	1,300	4.27 (3.95 – 4.59)	3.95 (3.66 – 4.23)	4.24 (3.94 – 4.55)	4.32 (4.00 – 4.63)
**Educational status of the mother**					
Low	476	3.40 (2.72 – 4.08)	3.27 (2.70 – 3.83)	3.61 (3.16 – 4.06)	3.32 (2.69 – 3.95)
Medium	3,362	3.73 (3.51 – 3.94)	3.76 (3.58 – 3.95)	3.94 (3.68 – 4.19)	3.97 (3.77 – 4.17)
Higher	1,254	4.44 (4.13 – 4.75)	4.11 (3.76 – 4.45)	4.33 (4.08 – 4.58)	4.31 (4.08 – 4.53)
**Number of siblings**					
0	2,169	3.90 (3.55 – 4.25)	3.63 (3.34 – 3.93)	3.99 (3.72 – 4.25)	3.87 (3.62 – 4.12)
1	1,850	3.43 (3.15 – 3.72)	3.86 (3.61 – 4.12)	3.94 (3.60 – 4.28)	3.83 (3.54 – 4.11)
2 or more	861	4.28 (3.93 – 4.64)	3.70 (3.11 – 4.29)	3.98 (3.61 – 4.35)	4.32 (3.88 – 4.75)
Twins/Multiple births	182	4.03 (2.80 – 5.26)	3.83 (3.02 – 4.64)	3.10 (1.90 – 4.30)	3.78 (2.69 – 4.87)
**Smoking in pregnancy**					
Yes	470	3.57 (2.88 – 4.27)	2.96 (2.19 – 3.73)	3.39 (2.52 – 4.26)	2.67 (2.06 – 3.27)
No	4,604	3.83 (3.58 – 4.04)	3.78 (3.58 – 3.97)	3.99 (3.78 – 4.19)	4.02 (3.85 – 4.19)
**(Pre)maturity**					
Premature birth	408	4.01 (3.46 – 4.57)	3.60 (3.01 – 4.18)	3.17 (2.44 – 3.90)	3.11 (2.44 – 3.78)
Mature or definitely post-term-birth	4,658	3.78 (3.57 – 3.99)	3.75 (3.56 – 3.95)	3.99 (3.79 – 4.19)	4.01 (3.83 – 4.19)
**Region**					
West	3,395	3.91 (3.69 – 4.14)	3.82 (3.62 – 4.02)	3.98 (3.78 – 4.23)	3.98 (3.78 – 4.19)
East (incl. Berlin)	1,700	3.35 (3.00 – 3.70)	3.41 (2.97 – 3.84)	3.68 (3.44 – 3.92)	3.79 (3.48 – 4.09)

M = mean, CI = confidence interval
